# Mouse Models for Exploring the Biological Consequences and Clinical Significance of *PIK3CA* Mutations

**DOI:** 10.3390/biom9040158

**Published:** 2019-04-23

**Authors:** Camilla B. Mitchell, Wayne A. Phillips

**Affiliations:** 1 Peter MacCallum Cancer Centre, Melbourne, VIC 3000, Australia; camilla.mitchell@petermac.org; 2 Sir Peter MacCallum Department of Oncology, The University of Melbourne, Parkville, VIC 3010, Australia

**Keywords:** PI3K, PI 3-kinase, PIK3CA, mouse model, knock-in, transgenic, cancer, overgrowth, PROS

## Abstract

The phosphatidylinositol 3-kinase (PI3K) pathway is involved in a myriad of cellular signalling pathways that regulate cell growth, metabolism, proliferation and survival. As a result, alterations in the PI3K pathway are frequently associated with human cancers. Indeed, *PIK3CA*—the gene encoding the p110α catalytic subunit of PI3K—is one of the most commonly mutated human oncogenes. *PIK3CA* mutations have also been implicated in non-malignant conditions including congenital overgrowth syndromes and vascular malformations. In order to study the role of *PIK3CA* mutations in driving tumorigenesis and tissue overgrowth and to test potential therapeutic interventions for these conditions, model systems are essential. In this review we discuss the various mouse models currently available for preclinical studies into the biological consequences and clinical significance of *PIK3CA* mutations.

## 1. Introduction

Phosphatidylinositol 3-kinases (PI3K) are a family of lipid kinases and can be divided into four classes (class IA, IB, II and III), based on structural characteristics [1]. Class IA PI3Ks are heterodimers composed of both a catalytic subunit (p110α, p110β or p110δ, encoded by the genes *PIK3CA*, *PIK3CB* and *PIK3CD*, respectively) and a regulatory subunit (p85α, p50α, p55α, p85β or p55γ, encoded by *PIK3R1*, *PIK3R2* and *PIK3R3*) [2,3,4]. Class IA PI3Ks are receptor activated and exert their effects by phosphorylating phosphatidylinositol-4,5-bisphosphate (PIP_2_) into phosphatidylinositol-3,4,5-trisphosphate (PIP_3_). Production of PIP_3_ activates downstream signalling hubs, such as AKT, which can go on to affect numerous downstream effectors involved in important cellular signalling pathways, including growth, metabolism, migration, proliferation and survival [3,5,6]. The levels of PIP_3_ are also regulated by PTEN, a lipid phosphatase which dephosphorylates the 3’ position of PIP_3_ converting it back to PIP_2_. 

Genomic alterations in the PI3K signalling pathway have been implicated in many different tumour types. The most common cancer-associated alterations in the PI3K pathway are activating mutations in *PIK3CA* which encodes p110α. *PIK3CA* mutations were first identified in 2004 [7,8] and are now known to occur at high frequencies in a wide range of solid tumours, including breast cancer (31%), endometrial cancer (37%), cervical cancer (29%), bladder cancer (22%), anal cancer (27%), colorectal cancer (17%) and head and neck squamous cell carcinoma (14%) [7,8,9,10,11,12,13,14]. The majority of cancer-associated *PIK3CA* mutations occur at three main hotspots in exon 9 (E542, E545) and exon 20 (H1047). These exons code for regions of the protein known to control enzymatic activity, and thereby to effect downstream targets of PIP3 [11,15]. 

*PIK3CA* mutations are also found in some non-malignant conditions. It was recently documented that postzygotic activating mutations in *PIK3CA* are present in several congenital malformation and overgrowth syndromes, including (but not limited to) congenital, lipomatous, overgrowth, vascular malformations, epidermal naevi and scoliosis (CLOVES) syndrome, fibroadipose hyperplasia, megalencephaly-capillary malformation syndrome, hemihyperplasia-multiple lipomatosis syndrome, hemimegalencephaly and facial infiltrating lipomatosis [16,17,18,19]. These conditions are now collectively known as *PIK3CA*-related overgrowth spectrum (PROS) [20]. PROS primarily affect children, manifesting at birth. Progressive overgrowth occurs throughout childhood resulting in segmental overgrowth of mesodermal tissues, typically affecting adipose tissue, fibroblasts, muscle and bone. However, the most common tissues affected are the vasculature (capillaries, veins and lymphatics) with most complications occurring due to vascular crises [20,21,22,23]. Somatic *PIK3CA* mutations have also been shown to underlie a proportion of congenital lymphatic and venous abnormalities [24,25]. Interestingly, although the specific mutations in *PIK3CA* are the same as those found in tumours, PROS and *PIK3CA*-driven vascular malformation conditions do not appear to be associated with an increased risk of cancer. PIK3CA somatic mutations have also been linked with epilepsy that arises through PIK3CA mutation driven developmental birth defects [26,27]. Interestingly this type of epilepsy does not respond to traditional seizure medications but there is evidence suggesting that targeting the PI3K pathway may reduce the incidence of seizures in PIK3CA driven epilepsy [27].

In order to study the role of *PIK3CA* mutations in human disease, a range of preclinical mouse models have been developed, including transgenic and conditional mutant mice. In addition to enabling in vitro and in vivo biological studies to assess the biochemical and clinical consequences of *PIK3CA* mutation, these models also allow the preclinical testing of potential therapeutic interventions for *PIK3CA*-associated conditions. In this review we discuss the preclinical mouse models of *PIK3CA* mutation that are currently available, with examples on how they have been used to further our knowledge of human disease. 

## 2. Modelling PIK3CA-Induced Cancers with Genetically Engineered Mouse Models 

Genetically engineered mouse models have enabled the expression of human mutations in the mouse and have allowed for massive increases in knowledge on which genes drive tumorigenesis and have proven that expression of mutations in these genes do indeed drive tumour development [28,29]. The discovery that *PIK3CA* mutations are involved in human cancers [7,8] has resulted in the development of multiple genetically engineered mouse models (Table 1) that have allowed for the role of *Pik3ca* mutations in cancer development and progression to be studied in detail and in multiple types of cancer.

### 2.1. Fusion Models

**Figure 1:**
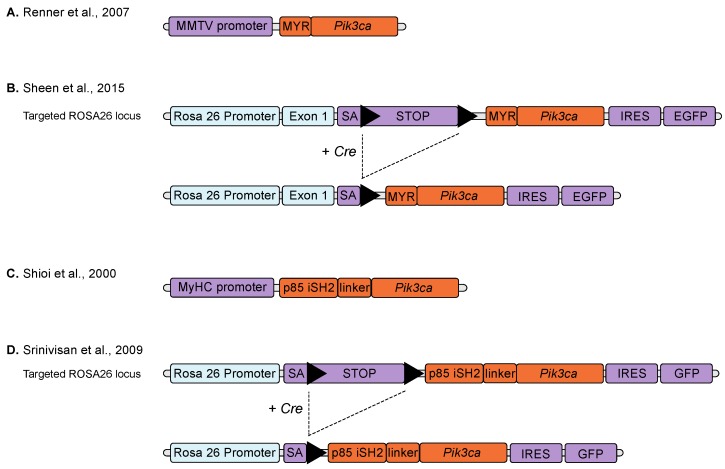
Schematic overviews of the transgenic p110α fusion models. (**a**) A simple transgene with a murine mammary tumour virus (MMTV) promoter upstream of a murine *Pik3ca* cDNA with a *Src* myristoylation sequence (MYR) added to the 5’-terminal end resulting in the expression of a myristoylated-p110α protein [30]. (**b**) A splice acceptor sequence (SA) and a *loxP*-flanked transcriptional stop cassette (STOP) upstream of a *Pik3ca* cDNA with a 5’-terminal myristoylation sequence (MYR), followed by an IRES-EGFP reporter element (an internal ribosome entry site (IRES) upstream of an enhanced green fluorescent protein (EGFP) cDNA), inserted into the ROSA26 gene locus. Upon expression of Cre recombinase (Cre) the stop cassette is excised allowing expression of the myristoylated-p110α and the EGFP reporter, under the control of the endogenous ROSA26 promoter [38]. (**c**) An α myosin heavy chain (MyHC) promoter-driven transgene containing a bovine *PIK3CA* construct with a 5’ sequence coding for the inter-SH2 domain (iSH2) of p85 separated by a glycine linker sequence, which generates a chimeric protein with p85 iSH2 fused to the N-terminus of p110α by a flexible linker (iSH2p110α*) [32]. (**d**) A splice acceptor sequence (SA) and a *loxP*-flanked transcriptional stop cassette (STOP) upstream of a p85 iSH2-linker-*PIK3CA* cDNA construct, followed by an IRES-GFP reporter element, inserted into the ROSA26 gene locus. Upon expression of Cre recombinase (Cre) the stop cassette is excised allowing expression of the iSH2p110α* protein and the GFP reporter, under the control of the endogenous ROSA26 promoter [33]. Triangles represent *loxP* sites. Blue colour indicates endogenous gene sequence, orange indicates modified *PIK3CA* constructs and purple indicates vector DNA sequence.

The earliest mouse models of *Pik3ca* activation used simple transgenic approaches. To investigate the effect of an enhanced PI3K activity in the epithelial cells of mammary glands, Renner et al. generated transgenic mice by injecting the cDNA of the murine p110α under the control of the epithelial-specific murine mammary tumour virus (MMTV) promoter into the pronucleus of single-cell mouse embryos [30,31]. To ‘activate’ the PI3K activity, the myristoylation sequence of src kinase was fused to the N-terminus of p110α (Figure 1A), leading to the localization of the p110α to the inner leaflet of the plasma membrane, resulting in the constitutive activation of its kinase activity. This mammary cell-targeted MYR-p110α model resulted in an increase in proliferative lesions in the mammary gland but did not progress to carcinoma [31]. Crossing MYR-p110α transgenic mice with heterozygous p53 knockout (p53^+/−^) mice did not change the mammary gland phenotype of the MYR-p110α mice nor increase tumorigenesis in the p53^+/−^ mice. However, the combination of MYR-p110α with a CDK4 activating mutation (R24C) led to increased tumorigenesis, demonstrating the interaction between the CDK4/Rb/E2F cascade and the PI3K signalling pathway observed in many human cancers [31].

This model was also used to study the effect of constitutive active p110α in the prostate gland. Transgenes under the control of the MMTV promoter are also known to be expressed in the male reproductive organs [69]. By identifying a mouse line in which the MYR-p110α transgene was integrated into the Y chromosome and thus only transmitted to male progeny, expression of the transgene and constitutive activation of the PI3K signalling cascade in the epithelial compartment of the prostate could be demonstrated [30].

Sheen et al. [38] also used the myristoylation approach, inserting a myristoylated form of p110α into the Rosa26 locus (Figure 1B). To avoid the embryonic lethality associated with constitutive *Pik3ca* activation during development, they used a Lox-STOP-Lox (LSL) cassette to make expression of the myristoylated allele conditional upon the presence of Cre-recombinase. In cells lacking Cre-recombinase, the LSL cassette blocks expression of MYR-p110α. However, in cells with active Cre-recombinase, Cre-mediated removal of the LSL cassette allows the expression of the MYR-p110α protein under the control of the endogenous Rosa26 promoter. Expression of the MYR-p110α in the mammary gland, induced by instillation of an adenovirus expressing Cre-recombinase into mammary ducts, by itself had minimal transforming activity but in an oncogenic mouse model with a homozygous p53 deletion and a *Kras*^G12D^ missense mutation, the addition of MYR-p110α accelerated mammary tumour initiation but not tumour growth rate [38].

A similar dominantly active PI3K model has been generated using iSH2p110*, a chimeric protein in which the iSH2 region of p85 is covalently linked via a flexible hinge region to its binding site at the N terminus of p110α (Figure 1C). Again, this was cloned behind a LSL cassette into the Rosa26 locus (Figure 1D) [33]. Crossing mice carrying this transgene with mice expressing Cre recombinase under the control of the fatty acid–binding protein promoter resulted in the expression of iSH2p110* in epithelial cells of the distal small bowel and colon [34]. After 40–60 days, mice became moribund due to large tumours in the proximal colon causing severe colon obstruction. These tumours penetrate through the serosa and contain enlarged blood vessels and lymphatic tissue [34]. This group has also used this model to demonstrate that expression of iSH2p110* synergizes with loss of APC activity resulting in increases in tumour number, size and invasiveness within the colon [35].

### 2.2. Mutation-Specific Transgenic Models

**Figure 2:**
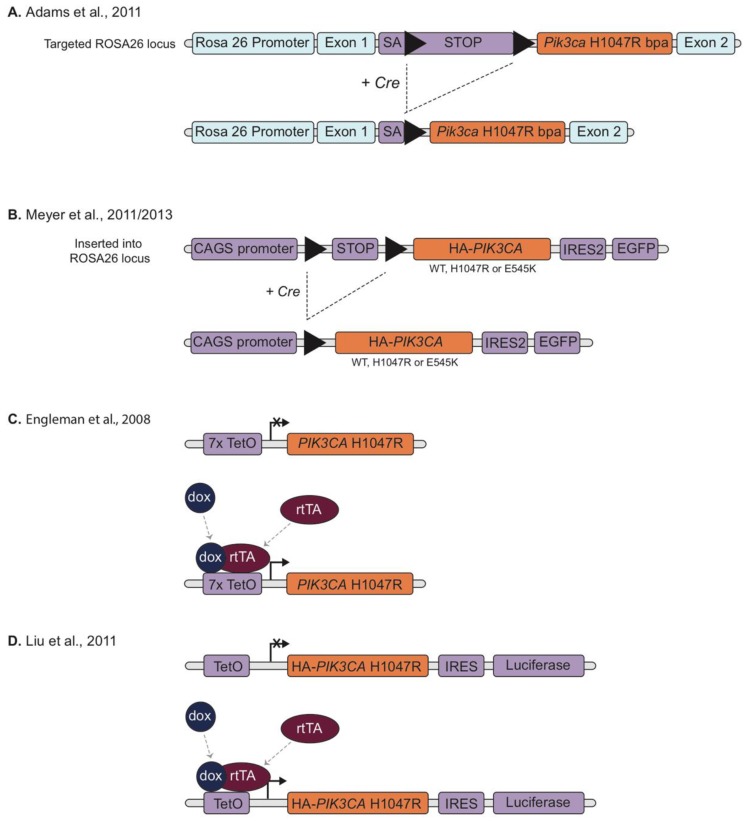
Schematic overviews of *Pik3ca* mutation specific transgenic models. (**a**) A mouse *Pik3ca* cDNA (modified to express the H1047R mutation), preceded by a splice acceptor sequence (SA) and a *loxP*-flanked transcriptional stop cassette (STOP), inserted into the ROSA26 gene locus. Upon expression of Cre recombinase (Cre) the stop cassette is excised allowing expression of mutant *Pik3ca* under the control of the endogenous ROSA26 promoter [40]. (**b**) A construct with a chicken β-actin (CAGS) promoter and a *loxP*-STOP-*loxP* sequence upstream of a 5’-terminally hemagglutinin (HA)-tagged human *PIK3CA* cDNA (wild type or modified to express H1047R or E545K mutations) and an *IRES2-EGFP* reporter element (an internal ribosome entry site (IRES) upstream of an enhanced green fluorescent protein (EGFP) cDNA), inserted into the ROSA26 locus. Expression of Cre recombinase results in the excision of the stop cassette allowing CAGS promoter-driven expression of the *Pik3ca* cDNAs and the green fluorescent protein (GFP) reporter [44,64]. (**c**) A DNA transgene consisting of seven direct repeats of the tetracycline operator (TetO) sequence followed by human *PIK3CA* cDNA containing a H1047R mutation. Reversible expression of the transgene requires the expression of a reverse tetracycline transactivator protein (rtTA) and is controlled by the administration of doxycycline (dox) [39]. (**d**) Similar to (C) but includes a downstream *IRES* and *luciferase* reporter gene [41]. Triangles represent *loxP* sites. Blue colour indicates endogenous gene sequence, orange indicates *Pik3ca* cDNA and purple indicates vector DNA sequence.

In order to model the effects of specific *PIK3CA* mutations, conditional knock-in mice were generated by cloning mutant *Pik3ca* cDNAs, preceded by a LSL cassette, into the Rosa26 locus. Both *Pik3ca* hotspot mutations *Pik3ca*^H1047R^ [40,44] and *Pik3ca*^E545K^ [44,64] have been modelled in this way (Figure 2A,B).

When expression of the transgenic allele was induced by Cre recombinase driven by the mammary restricted promotor MMTV, Adams et al. found that mice expressing *Pik3ca*^H1047R^ started developing mammary tumours (typically adenosquamous carcinoma or adenomyoepithelioma) at 5 months of age with 69% of mice developing palpable mammary tumours at end point (the remaining mice had to be sacrificed due to development of lymphoma/thymoma, skin and other non-mammary tumours which was attributed to Cre expression in other tissues) [40]. Similar results were observed by Meyer et al. although in their model approximately 75% of the MMTV-Cre:*Pik3ca*^H1047R^ animals died before the age of 4 months, presumably due to leakiness of the MMTV promoter causing deleterious *Pik3ca*^H1047R^ expression in tissues other than the mammary gland [44]. Adams et al. also tested for a genetic interaction between *Pik3ca*^H1047R^ and p53 loss-of-function mutations. This led to decreased survival of double-mutant animals, which developed lymphoma and mammary tumours with rapid kinetics [40]. 

Meyer et al. also used a Cre recombinase driven by the WAPi promoter (Whey Acidic Protein; active in mammary alveolar progenitor cells and differentiated secretory luminal cells) in this model [44]. WAPi-cre:*Pik3ca*^H1047R^ mice developed mammary tumours at an average age of around 140 days. They also observed pregnancy accelerated tumour initiation, most likely due to an increase in the number of cells expressing the mutation [44]. Of note, overexpression of wild type *Pik3ca* was insufficient to induce mammary tumours after 520 days [40].

Meyer et al. also generated transgenic mice expressing the *Pik3ca*^E545K^ mutation. Parous WAPi-Cre:*Pik3ca*^E545K^ mice developed mammary tumours 80 days after delivery, which was a longer latency than that observed in WAPi-Cre:*Pik3ca*^H1047R^ mice [44,64]. 

*Pik3ca*^H1047R^ overexpression was also used to determine lineage effects in mammary tumours by specifically targeting the mutation to either basal (LGR5 positive) or luminal (keratin 8 positive) lineage restricted cells (using Lgr5-CreER^T2^ and K8-CreER^T2^, respectively) resulting in heterogeneous, multi-lineage mammary tumours. This work demonstrated the effect of *PIK3CA*^H1047R^ on mammary cell fate in the pre-neoplastic mammary gland and revealed that the cell of origin of *PIK3CA*^H1047R^ tumours dictates their malignancy [45]. 

### 2.3. Reversible Models

Overexpression models of *PIK3CA*^H1047R^ mutations have been developed using the reversible tetracycline-controlled transactivator (rtTA) system. The rtTA dependent system allows for reversible gene expression as the presence of tetracycline (or an analogue, such as doxycycline) is required for promoters to drive transgene gene expression. This approach can be combined with tissue specific promoters to provide both spatial and temporal control over expression of the transgene.

Engelman et al. produced transgenic mice with a tetracycline-inducible expression of human *PIK3CA^H1047R^* (*rtTA-Tet-op-PIK3CA^H1047R^*) using a construct consisting of seven direct repeats of the tetracycline (tet)-operator sequence followed by *hPIK3CA^H1047R^* cDNA (Figure 2C). Mice expressing the *Tet-op-hPIK3CA^H1047R^* transgene were then crossed to mice in which the expression of the reverse tetracycline trans-activator protein (rtTA*)* was driven by the Clara Cell Secretory Protein (CCSP) promoter (targets type II alveolar epithelial cells) to generate inducible, bitransgenic mice harbouring both activator and responder transgenes [39]. Adenocarcinoma could be detected in the lungs of induced mice within 6 weeks of doxycycline administration. Subsequent withdrawal of doxycycline resulted in a loss of mutant *PIK3CA* expression and led to rapid and dramatic tumour regression, thereby demonstrating that maintenance of the established lung tumours required continued expression of p110α^H1047R^. A similar regression of the tumours was observed after treatment of mice with the dual PI3K/mTOR inhibitor NVP-BEZ235 but not with the mTOR inhibitor rapamycin [39].

Liu et al. [41] used a similar approach to study the effects of mutational activation of PI3K on breast tumorigenesis. They generated a bitransgenic mouse line expressing human *PIK3CA^H1047R^* under control of a tetracycline-inducible promoter (rtTA-TetO-HA-*PIK3CA*^H1047R^-IRES-luciferase) and rtTA under control of the MMTV promoter (MMTV-rtTA) to drive mammary-specific expression of *PIK3CA^H1047R^* (Figure 2D). Female mice treated with doxycycline showed increased mammary ductal side-branching and enlarged focal nodular structures filled with hyperproliferative cells characteristic of early neoplastic lesions at 4 weeks and went on to develop mammary tumours with heterogeneous pathological phenotypes, including adenocarcinomas and adenosquamous carcinomas, with 95% penetrance and a mean latency of 7 months [41]. Although a proportion of the tumours completely or partially regressed following doxycycline removal and did not resume growing, about two-thirds of the tumours initially regressed but then resumed growth in the absence of doxycycline while maintaining sustained downregulation of the *PIK3CA^H1047R^* transgene and its protein product [41].

This model has also been used to demonstrate that HER2 and mutant *PIK3CA* cooperate to promote mammary tumour establishment and metastatic progression [43]. These double mutant tumours are resistant to inhibitors of HER2 (viz. trastuzumab alone and in combination with lapatinib or pertuzumab). This resistance could be reversed by the PI3K inhibitor BKM120, suggesting that anti-HER2 therapies together with PI3K inhibitors may be a beneficial combination for the clinical treatment of HER2^+^/*PIK3CA*-mutant breast cancers [43].

While the transgenic mouse models described above have proven very valuable, they do have a number of significant limitations. Importantly, expression of the transgene is invariably driven by exogenous promoters and thus is not subject to normal regulatory mechanisms that control expression of the endogenous gene. As a result, the transgene may be expressed at non-physiological levels or even in cells that do not normally express *Pik3ca*. In addition, the transgene is expressed in a background of normal expression of the endogenous gene.

### 2.4. Conditional Knock-In Models

In contrast to the standard transgenic approach, knock-in mouse models allow for specific mutations to be introduced into endogenous *Pik3ca* gene and carried through the germline so that the expression of the mutant is under control of the endogenous *Pik3ca* promoter and normal regulatory mechanisms. 

**Figure 3:**
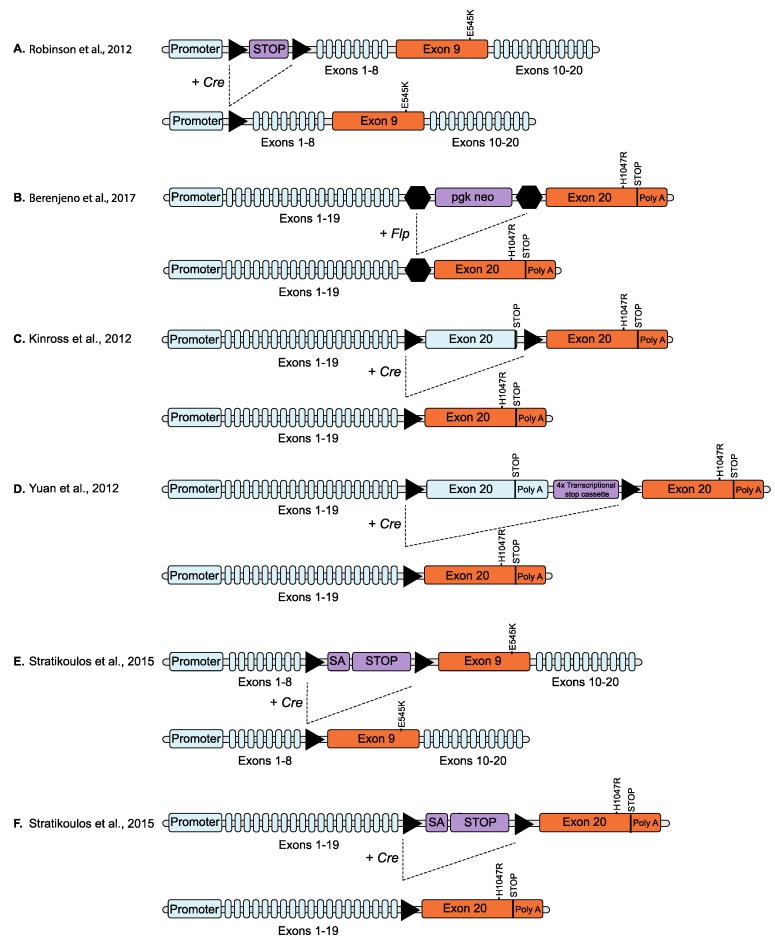
Schematic overviews of conditional knock-in PIK3CA mutation models. Triangles represent loxP sites. (**A**) Homologous recombination was used to insert a *loxP*-flanked transcriptional stop cassette (STOP) immediately upstream of the exon containing the initiation codon and replace exon 9 with an exon containing a E545K mutation, in one allele of the endogenous *Pik3ca* gene. Expression of the mutant allele is prevented by the stop cassette until removed by the expression of Cre recombinase (Cre) [63]. (**B**) A neomycin selection cassette (pgk neo) flanked by *frt* sites was inserted in the intron between exons 19 and 20 and point mutations resulting in a H1047R mutation introduced into exon 20, of one allele of the endogenous *Pik3ca* gene. The presence of the neo cassette suppresses the expression of the targeted allele until removed by Flp recombinase [65]. (**C**) *LoxP* sites were inserted on either side of the endogenous wild type exon 20 and a copy of exon 20 coding for a H1047R mutation inserted downstream of the endogenous stop codon (and after the downstream *LoxP* site) in one allele of the endogenous *Pik3ca* gene. The modified allele continues to express wild type protein (with expression of the inserted mutant exon prevented by the stop codon in the endogenous exon 20) until the expression of Cre recombinase which removes the wild type exon 20, replacing it with the mutated exon 20 [46]. (**D**) Similar to (C) but includes a transcriptional stop cassette downstream of the endogenous gene and before the inserted mutant exon [65]. (**E**) Point mutations coding for a E545K mutation were introduced into exon 9 of one allele of the endogenous *Pik3ca* gene and a *loxP*-flanked splice acceptor site (SA) and transcriptional stop cassette inserted into the intron immediately upstream of the mutated exon. Expression of the mutated exon is prevented by the stop cassette until removed by Cre recombinase [67]. (**F**) Similar to (E) but targeting exon 20 (H1047R) [68]. Triangles represent *loxP* sites and hexagons represent *frt* sites. Blue colour indicates endogenous gene sequence, orange indicates modified *Pik3ca* exon and purple indicates vector DNA sequence.

Unfortunately, germline expression of oncogenic *Pik3ca* mutations is embryonic lethal [62], consequently a conditional knock-in is required to enable normal development of the mouse. One successful approach inserted a LSL cassette immediately upstream of the initiation codon of one allele of the endogenous *Pik3ca* gene and exon 9 of the same allele was replaced with an exon containing the E545K mutation (Figure 3A). The stop codon in the LSL cassette prevents expression of the modified allele and so only the wild type allele is expressed but, in the presence of Cre recombinase, the LSL cassette is removed allowing the expression of the mutated (*Pik3ca*^E545K^) allele [63]. These mice were crossed with mice harbouring Cre-conditional loss of *Tp53* (*Tp53^+/flx^* or *Tp53^flx/flx^*) and/or *Ctnnb1* (*Ctnnb1^+/lox(Ex3)^*) and a *Blbp-Cre* transgene (expresses Cre recombinase in progenitor cell populations across the hindbrain). *Pik3ca*^E545K^ expressing mice, with or without homozygous loss of *Tp53*, survived tumour free for a median of 212 days. However, 100% of triple mutant mice, with *Pik3ca*^E545K^ and heterozygous loss of both *Tp53* and activation of *Ctnnb1* through in-frame deletion of exon 3, developed WNT-subgroup medulloblastomas by 3 months of age, compared with only 4% of the *Blbp-Cre:Ctnnb1^+/lox(Ex3)^*:*Tp53^+/flx^* controls developing tumours by 11 months [63].

A similar approach was used by Berenjeno et al. [66] who inserted a H1047R mutation into one allele of the endogenous *Pik3ca* gene with the presence of a neomycin (*Neo*) selection cassette in the targeted *Pik3ca* locus, suppressing the expression of the modified allele to allow normal embryonic development. In this case, expression of *Pik3ca*^H1047R^ is achieved by removal of the *Neo* cassette, through recombination via its flanking frt sites, mediated by tamoxifen-induced activation of a Flp recombinase transgene (*CAG::Flpe-ER*^T2^)(Figure 3B). They used this model to demonstrate that mutant *Pik3ca* induces centrosome amplification, tolerance to tetraploidization and induction of aneuploidy. Consistent with previous data suggesting that centrosome amplification on its own is not an independent driver of cancer development [70,71], induction of *Pik3ca*^H1047R^ alone in this model did not produce neoplastic lesions or cancer, as assessed in multiple organs but accelerated the onset of cancer when combined with intestine-specific heterozygous deletion of the *Apc* tumour suppressor gene (*Apc*^flox/+^ mice) [66].

The most widely used and arguably the most physiologically relevant, model of *Pik3ca* mutation is the model generated by Kinross et al. [46]. Taking advantage of the fact that the site of the H1047R mutation is in exon 20, the very last exon of the endogenous gene, they have used a novel ‘exon-swap’ strategy to conditionally knock in the H1047R mutation into the endogenous *Pik3ca* gene. LoxP sites flanking the wild type exon 20 were inserted into one allele of *Pik3ca* and a tandem copy of exon 20 containing the H1047R mutation placed downstream of the wild type exon. Prior to Cre-mediated recombination, normal wild type *Pik3ca* is expressed from this allele, while the introduction of Cre results in the deletion of the wild type exon 20 and replaces it with the mutant version (Figure 3C). This results in an inducible *Pik3ca*^H1047R^ mutation that is expressed at physiological levels and only in cells that would normally express *Pik3ca*, thus accurately mimicking the scenario of a heterozygous somatic mutation in the endogenous gene, as occurs in human tumours [46]. 

The initial description of this model used an intrabursal delivery of an adenovirus expressing Cre recombinase to activate expression of the *Pik3ca*^H1047R^ mutation in the mouse ovary. This resulted in premalignant hyperplasia of the ovarian surface epithelium but no tumours. However, induction of the *Pik3ca*^H1047R^ mutation plus homozygous Pten deletion (*Pten*^flox^ mice) in the mouse ovary led to the development of ovarian serous adenocarcinomas and granulosa cell tumours [46]. 

Subsequent studies have used a wide range of Cre recombinase transgenes with this model to specifically target *Pik3ca*^H1047R^ expression to various tissues including mammary gland [47,48], intestinal tract [58], lung [49,50], melanocytes [51,52], prostate [59], brain [60], pancreas [53], thyroid [54,55,56] and epidermis [57], as well as more generalised expression approaches [25,61,62]. The general theme arising from these studies is that, apart from rare exceptions, *Pik3ca*^H1047R^ expressed alone at physiological levels does not induce spontaneous tumorigenesis but rather cooperates with mutations in other oncogenes such as *BRaf* [50,52,54] and *KRas* [49] or with loss of tumour suppressors genes including *Pten* [46,59,60], *Tp53* [48] and *Apc* [58], to enhance the initiation and/or progression of tumorigenesis. 

The failure of *Pik3ca*^H1047R^ alone to induce tumours in this model (and also in the knock in models of Robinson [63] and Berenjeno [66]) is different from the transgenic mouse models driven by exogenous promoters, where *Pik3ca* mutation alone was able to induce lung, breast and colon tumours [34,39,40,41,44]. The exceptions to this are the mammary gland where tumours were seen with *Pik3ca*^H1047R^ alone in the knock-in model [47,48], albeit with longer latency than similar transgenic models [40,41,44] and the prostate where *Pik3ca*^H1047R^ alone resulted in locally invasive prostate carcinoma, again with long latency (300 to 400 days) [59]. Nevertheless, in both these cases, *Pik3ca*^H1047R^ was found to synergise with other mutations (*Tp53* loss in mammary gland [48] and *Pten* loss in prostate [59]) to greatly accelerate tumorigenesis.

Another knock-in model, using a similar ‘exon-swap’ approach to Kinross et al., has been independently reported by Yuan et al. [65] (Figure 3D). In their model they used a MMTV-driven Cre recombinase to induce expression of the *Pik3ca*^H1047R^ knock-in in the mammary gland. Consistent with the results of Tikoo et al. [47,48], Yuan et al. found that activation of the latent *Pik3ca*^H1047R^ allele resulted in mammary tumours with multiple histological types but with a long latency. Interestingly, whole-exome analysis of the *Pik3ca*^H1047R^-driven mammary tumours identified multiple other somatic mutations, including *Tp53* mutations, that appeared spontaneously during tumour development [65]. This would be consistent with *Pik3ca*^H1047R^ requiring an additional ‘second hit’ mutation in another gene in order to initiate tumorigenesis which may explain the long latency observed with *Pik3ca*^H1047R^ alone in knock-in models. 

Stratikopulous et al. [67] also generated Cre-conditional *Pik3ca* hotspot mutant mice (both E545K and H1047R) and crossed these with MMTV-Myc mice to initiate mammary tumorigenesis and WAP-Cre mice to drive *Pik3ca* mutant expression in the mammary gland (Figure 3E,F). Since the WAP promoter is endogenously activated at a later time point to MMTV, this effectively results in a “two-hit” model in which mammary tumorigenesis is first initiated by Myc expression followed by the subsequent induction of the *Pik3ca* mutation. When compared to *Myc* overexpressing females, double mutants (both MMTV-*Myc;Pik3ca*^E545K/+^;*WAP*^Cre^ and MMTV-*Myc*;*Pik3ca*^H1047R/+^;*WAP*^Cre^) develop focal mammary tumours much quicker, within 1 week of the first parturition, compared to 115 days after first parturition [67]. This model provides evidence that *Pik3ca* mutations are involved in the progression of tumorigenesis. This group also used this same model (H1047R) driven by WAP-Cre, in combination with exogenous administration of oestrogen to drive ER+, *Pik3ca*^H1047R^ mutant mammary tumours [68]. Mammary tumours with 100% penetrance developed 353 days after first parturition in *Pik3ca* mice with oestrogen administration, compared with tumour development at 471 days after first parturition (with 68% penetrance) in *Pik3ca*^H1047R^ mutants without oestrogen administration [68].

## 3. Mouse Models of Non-Malignant *PIK3CA*-Related Conditions

While the majority of mouse models of *PIK3CA* mutation have focused on tumorigenesis, these models also allow for the study of other pathogenic effects of *PIK3CA* mutation that are not related to cancer. Indeed, activating mutations in *PIK3CA* have been linked to a spectrum of noncancerous overgrowth disorders [16,17,18,19] (collectively known as *PIK3CA*-related overgrowth spectrum (PROS) [20] and isolated (not associated with overgrowth) vascular (lymphatic [18] and venous [24,25]) malformations. Interestingly, although present from birth, the *PIK3CA* mutations found in PROS and vascular malformations are similar to those found in solid tumours, with the H1047R mutation being the most common but occur in a mosaic pattern, mainly in tissues of mesodermal origin [18,20]. This contrasts with somatic *PIK3CA* mutations in cancer, which are almost exclusively present in epithelial tissues. 

One of the earliest mouse models of a constitutive active *PIK3CA* was generated by Shioi et al. to study the role of PI3K in organ size. They used a transgene expressing the iSH2p110* fusion protein under the control of the α myosin heavy chain (MyHC) promoter (Figure 1C) to demonstrate that constitutive activation of *PIK3CA* in cardiac myocytes results in an increase in heart size [32]. 

Sheen et al. utilised a CMV Cre-inducible MYR-p110α to target p110α to the membrane, thereby activating it [37]. When activated early in embryogenesis, most cells gained genomic alterations causing embryonic lethality. Most abnormalities were associated with incorrectly developed vasculature, undefined blood vessels, haemorrhaging, lack of normal blood circulation and defective vasculogenesis and angiogenesis, suggesting that over activation of PI3K signalling disrupts normal vascular development [37]. 

In order to explore the potential pathogenic effects of wide-spread expression of *PIK3CA* mutation, Hare et al. used the exon-swap *Pik3ca*^H1047R^ knock-in mouse crossed with the Cre-deleter (*CMV-cre*) transgenic mouse to induce ubiquitous expression of *Pik3ca*^H1047R^ from the early zygote stage of development. This led to multiple severe developmental abnormalities, including vascular defects as well as impaired haematopoiesis, resulting in embryonic lethality [62]. This finding is consistent with the lack of germline *PIK3CA* mutations in cancer patients and the observation that *PIK3CA* mutations associated with *PIK3CA*-related overgrowth spectrum (PROS) are acquired post-zygotically and are not inherited [16,17,19,20,72] and demonstrates that activating mutations in *PIK3CA* are not compatible with germline transmission. To overcome this limitation, Kinross et al. used a tamoxifen-inducible Cre recombinase driven by the ubiquitin C promoter (*UCre*^ERT2^) to induce widespread expression of *Pik3ca*^H1047R^ in adult mice. This led to an unexpected early death, coinciding with widespread increases in organ size, hypoglycaemia, intolerance to fasting and increased glucose turnover [61], highlighting the critical role of *PIK3CA* in metabolism and growth. Similar early death upon induction of a transgenic LSL-*Pik3ca*^H1047R^ in widespread tissues of adult mice (using a tamoxifen-inducible chicken beta actin promoter driven Cre (*CAGG-Cre*^ER^)) has also been reported and, in this case, attributed to vascular defects [24,36]. 

Since PROS results in segmental overgrowth of mesodermal tissues, Castillo et al. used a tamoxifen–inducible Cre recombinase under the control of the Brachyury transcription factor promoter (*T-Cre*^ERT2^) and low doses of 4-hydroxytamoxifen, to activate *Pik3ca*^H1047R^ in a mosaic fashion in the embryonic mesoderm between embryonic days 7.5–10.5. These mice were born with subcutaneous vascular malformations in different body locations, reminiscent of PROS segmental overgrowth, however no overgrowth developed [25]. This phenotype was reminiscent of venous abnormalities seen in humans leading Castillo et al. to examine human venous malformations for PIK3CA mutations. Indeed, 23% of the human venous malformations tested were found to harbour *PIK3CA* mutations [25]. Similar results have also been reported by Castel et al. [24].

Consistent with these findings, when *Pik3ca*^H1047R^ was induced embryonically in endothelial cells (driven by *Tie2-Cre*), embryonic lethality occurred, largely due to vascular defects including disorganised and truncated vascular networks and impaired haematopoiesis [24,45,72]. Similarly, when *Pik3ca*^H1047R^ was induced in endothelial cells postnatally at P1 (driven by *Pdgfb-iCre*^ER^), mice developed hyperproliferation of retinal endothelial cells, loss of arteriovenous identity markers and loss of pericytes [25]. To investigate these vascular defects further, VE-Cadherin promoter (*Cdh5-Cre*^ERT2^) was used to drive expression of *Pik3ca*^H1047R^ in vascular endothelial cells at 8–10 weeks of age, resulting in 100% mortality within 15 days. Mortality was due to cardiac degeneration and vacuolated cardiomyocytes [42]. When activated intramuscularly, *Pik3ca*^H1047R^ expression induced vascular malformation and bleeding [42].

*PIK3CA*-related segmental overgrowth also occurs within the brain, resulting in bilateral dysplastic megalencephaly, hemimegalencephaly and focal cortical dysplasia [19,73,74]. Using both transgenic overexpression of *Pik3ca*^H1047R^ [41] and endogenous knock-in of *Pik3ca*^E545K^ [63] models, crossed with developing neural progenitor promoters (Nestin-Cre, Nestin-creERT2, hGFAP-Cre), all key human pathological features were represented, including brain enlargement, cortical malformation, hydrocephalous and epilepsy [27]. Interestingly, phenotypic severity appears to be dependent on the mutant allele, with H1047R causing more severe phenotypes than E545K, although the two mutations were expressed using different approaches. Additionally, developmental timing of mutational activation affected phenotypes, with embryogenic activation causing more severe phenotypes than postnatal activation. 

Recently, Venot et al. generated a mouse model of PROS using a chimeric *PIK3CA* transgene (R26StopFLP110*) with *CAGG-Cre*^ER^ to generate mice that ubiquitously express a dominant active PIK3CA (iSH2p110*) upon tamoxifen administration [36]. Using a high dose of tamoxifen (40 mg kg^−1^) in 3 week old mice resulted in sudden death with 50% of mortality within 9 days of administration, a result similar to a previous study [61]. However, by reducing the dose of tamoxifen (4 mg kg^−1^) to induce a lower rate of mosaicism, the mice survived for two months before dying with multiple phenotypic abnormalities, including organomegaly, progressively developed asymmetrical overgrowth of extremities, disseminated voluminous tumours and visible subcutaneous vascular abnormalities, similar to the lesions observed in patients with PROS [36]. This model was then used to demonstrate the efficacy of a PI3K inhibitor, BYL719, in treating these lesions. The success of these preclinical studies led to a clinical trial in human PROS patients with all 17 patients exhibiting a substantial clinical improvement following BYL719 treatment [36]. 

## 4. Concluding Remarks

*PIK3CA* is one of the most commonly mutated human oncogenes. *PIK3CA* mutations have also been implicated in non-malignant conditions including congenital overgrowth syndromes and vascular malformations. However, the precise molecular and cellular consequences of mutating the *PIK3CA* gene and how they drive clinical phenotypes, are not fully understood. 

In order to study the physiological role of *PIK3CA* mutations, there are a number of different approaches to model *PIK3CA* mutation in mice. These range from simple transgenic overexpression of mutant *PIK3CA* cDNAs to more sophisticated models that knock the mutations into the endogenous *Pik3ca* gene. Each has its advantages and disadvantages; while some of the transgenic models have the benefit of being reversible, the knock-in models, while irreversible, are arguably more physiologically relevant and more accurately reproduce the scenario of a somatic mutation as occurs in humans. 

These mouse models provide a significant opportunity to study the effects of *PIK3CA* mutations *in vivo* in a physiologically relevant context. While initial studies have been illuminating, the full potential of these models in unravelling the biological and clinical consequences of *PIK3CA* mutation is still to be revealed. 

## Figures and Tables

**Table 1:** Original mouse models of *PIK3CA* mutations.

Original Ref	Mouse Model	Genetic Approach	Inducible?	Target Tissue (Promoter)
Shioi et al., 2000 [32]	iSH2p110α*	Transgenic	Non-inducible	Cardiac myocytes (αMyHC) [32]
Srinivasan et al., 2009 [33]	iSH2p110α*	Transgenic	Non-inducible	Mature B cells (CD21) [33]
				Distal small bowel epithelial cells (Fatty acid binding protein) [34,35]
				All cells (CAGG) [36]
Renner et al., 2007 [30]	*MYR-p110α*	Transgenic	Non-inducible	Prostate epithelial cells (MMTV) [30]
				Mammary epithelial cells (MMTV) [31]
				All cells, early embryos (CMV) [37]
				Mammary duct (adenoviral) [38]
Engleman et al., 2008 [39]	*rtTA-Tet-op-PIK3CA^H1047R^*	Transgenic	Tetracycline inducible (doxycycline)	Type II alveolar epithelial cells (CCSP) [39]
Adams et al., 2011 [40]	*Cre^NLST^;* *Pik3ca^H1047R^*	Transgenic	Non-inducible	Mammary epithelial cells (MMTV) [40]
				Luminal and glandular uterine epithelial cells (Sprr2f); All cells (UBC); Blood vessels (Tie2) [24]
Liu et al., 2011 [41]	*rtTA TetO-Pik3ca^H1047R^*	Transgenic	Tetracycline inducible (doxycycline)	Mammary epithelial cells (MMTV) [41]
				Blood vessels (Tie2); VE-cadherin (Cdh5) [42]
				Mammary epithelial cells (MMTV) [43]
Meyer et al., 2011 [44]	*Cre; Pik3ca^H1047R^*	Transgenic	Non-inducible	Mammary epithelial cells (MMTV); Alveolar progenitor cells (WAPi) [44]
				Neural progenitor cells (hGFAP) [27]
				Basal and luminal mammary epithelium cells (Lgr5, K8) [45]
Kinross et al., 2012 [46]	*Cre; Pik3ca^H1047R^*	Knock-in	Oestrogen Receptor inducible (Tamoxifen)	Ovarian bursal cells (adenoviral) [46]
				Mammary epithelial cells (MMTV, K5, K8) [47,48]
				Lung (adenoviral) [49,50]
				Melanocytes (Tyrosinase) [51,52]
				Pancreatic cells (p48, Pdx1) [53]
				Thyroid (thyroglobulin) [54,55,56]
				Epidermis (lentivirus) [57]
				Intestinal epithelial cells (Gpa33) [58]
				Prostate cancer (probasin) [59]
				Neural stem/progenitor cells (nestin) [60]
				Embryonic mesoderm (T gene); Endothelial cells (Pdgfb) [25]
				All cells (UBC) [61]
				Endothelial cells (Tie2) [62]
Robinson et al., 2012 [63]	*Cre;* *Pik3ca^E545K^*	Knock-in	Inducible	Lower rhombic lip progenitor cells (Blbp) [63]
				Neural progenitor cells (hGFAP, Nestin) [27]
Meyer et al., 2013 [64]	*Cre; Pik3ca^E545K^*	Transgenic	Non-inducible	Alveolar progenitor cells (WAPi) [64]
Yuan et al., 2013 [65]	*Cre; Pik3ca^e20H1047R^*	Knock-in	Inducible	Mammary epithelial cells (MMTV) [65]
Berenjeno et al., 2017 [66]	*Flpe-ER^T2^;* *Pik3ca^H1047R^*	Knock-in	Tamoxifen Inducible	All cells (CAG) [66]
Stratikopoulos et al., 2015 [67]	*Myc; Pik3ca^E545K^* *Myc; Pik3ca^H1047R^*	Knock-in	Non-inducible	Mammary epithelial cells (MMTV, WAP) [67,68]
